# Dysmenorrhea and Its Impact on Patients’ Quality of Life—A Cross-Sectional Study

**DOI:** 10.3390/jcm13195660

**Published:** 2024-09-24

**Authors:** Mihaela Amza, Sebastian Findeklee, Bashar Haj Hamoud, Romina-Marina Sima, Mircea-Octavian Poenaru, Mihai Popescu, Liana Pleș

**Affiliations:** 1 Department PhD, IOSUD, “Carol Davila” University of Medicine and Pharmacy, 020021 Bucharest, Romania; mihaela.amza@umfcd.ro; 2 Department of Obstetrics and Gynecology, “Carol Davila” University of Medicine and Pharmacy, 020021 Bucharest, Romania; mircea.poenaru@umfcd.ro (M.-O.P.); liana.ples@umfcd.ro (L.P.); 3 “Bucur” Maternity, Saint John Hospital, 012361 Bucharest, Romania; 4 Department of Obstetrics and Gynecology, Faculty of Medicine, Saarland University Hospital, 66424 Homburg, Germany; sebastian.findeklee@uks.eu (S.F.); bashar.hajhamoud@uks.eu (B.H.H.); 5 Department of Anaesthesia and Intensive Care, “Carol Davila” University of Medicine and Pharmacy, 020021 Bucharest, Romania; mihai.popescu@umfcd.ro; 6 Department of Anaesthesia and Intensive Care, Bucharest University Emergency Hospital, 050098 Bucharest, Romania

**Keywords:** dysmenorrhea, quality of life, menstrual pain, DysmenQoL score

## Abstract

**Background:** Dysmenorrhea is a common condition that may have negative effects on social life, couples’ relationships and professional activities. The objectives of this study were to evaluate the prevalence, risk factors and characteristics of dysmenorrhea and its impact on patients’ quality of life using a specific self-questionnaire named “DysmenQoL questionnaire”. We also checked the validity and reliability of this questionnaire in our population. **Methods:** We conducted a cross-sectional study that included 504 participants of reproductive age between 18 and 45 years of age. The data were collected with an original form divided into three sections. The last section (DysmenQoL questionnaire) included 20 statements scored from 1 (“never”) to 5 (“every time”) that evaluates the effects of menstrual pain on health and feelings, daily activities, relationships and professional activity. We calculated the sum of the scores for each statement and we called it the “DysmenQoL score”. **Results:** The prevalence of dysmenorrhea was 83.7%. The presence of dysmenorrhea was statistically significant associated with the degree of menstrual bleeding (*p* = 0.017), the presence of infertility (*p* = 0.034) and dyspareunia (*p* = 0.002), but also with the presence of premenstrual syndrome and a family history of dysmenorrhea (*p* < 0.001). Among the participants with dysmenorrhea, 73.9% considered that this symptom affected their quality of life, and this was correlated with pain intensity and the DysmenQoL score (*p* < 0.001). A significant difference regarding the DysmenQoL score depending on the pain intensity, frequency and duration of dysmenorrhea and the methods used to reduce the pain was observed. **Conclusions:** Dysmenorrhea had a high prevalence among the participants included in the study, and its presence was associated with a series of risk factors. Most women considered that dysmenorrhea affected their quality of life. The DysmenQoL questionnaire proved to be a reliable and valid method for evaluating the impact of dysmenorrhea on quality of life.

## 1. Introduction

Dysmenorrhea was defined as the presence of painful menstruation and its prevalence was reported to vary between 16 and 91%, with severe pain occurring in 2% to 29% of cases [1,2]. A higher rate of dysmenorrhea was observed among adolescents between 12 and 19 years old [3,4]. Two types of dysmenorrhea have been described: primary and secondary dysmenorrhea. Most of the time, primary dysmenorrhea appears at 6 months after menarche, and it has no identifiable cause. Usually, the pain is more intense in the first two days of menstruation, lasts between 8 and 72 h, and can be associated with numerous other symptoms such as fatigue, dizziness or insomnia [5]. The occurrence mechanism involves the cyclooxygenase pathway and the production of prostaglandins that can cause the contraction of the uterine muscles with the reduction of blood flow and the stimulation of pain receptors by anaerobic metabolites [6]. Secondary dysmenorrhea usually occurs more than two years after menarche and it is associated with pelvic diseases such as endometriosis, uterine fibroids, ovarian cysts, uterine polyps or adenomyosis [7].

Several risk factors for dysmenorrhea have been identified, including heavy menstrual flow, family history of dysmenorrhea [8], the presence of premenstrual syndrome [9] (affective, cognitive or somatic symptoms that occur before menstruation, such as increased appetite, constipation, nausea, weight gain, headache, breast tenderness, fatigue, restlessness, anxiety or mood changes) [10], young age of menarche, nulliparity, low intake of omega 3 fatty acids [5] and smoking or regular coffee consumption [6]. It has been observed that maintaining a normal body mass index and reduction of stress can be beneficial in preventing dysmenorrhea [11]. It was found that the onset and frequency of dysmenorrhea in adolescence are predictive factors for the presence of endometriosis in adulthood [12].

It was observed that dysmenorrhea influenced women’s daily activities and negatively affected their quality of life [13]. Among female students, the presence of dysmenorrhea had negative effects on academic performance due to the appearance of difficulties in studying, the impossibility to concentrate in classes [14] and absenteeism, but also sleep disturbances or problems in personal relationships [15].

Most of the time, dysmenorrhea is not properly managed so menstrual pain persists and can have negative effects on women’s quality of life. Sometimes dysmenorrhea is not considered important; in addition, women can believe that it represents a normality. Women with dysmenorrhea have a low quality of life for several days each month and they have a poorer mood [16]. The most effective drugs prescribed to reduce pain are non-steroidal anti-inflammatory drugs (NSAIDs) [17]. Apart from pharmacological agents, complementary and alternative medicine is used to relieve menstrual pain [18]. Among the non-pharmacological methods, warming techniques such as hot packs, hot water or clothes have been identified as the most used to reduce menstrual pain [19]. It was found that practicing yoga for 30 min twice a week for 12 weeks can relieve menstrual pain [20].

The purpose of this study was to evaluate the characteristics of dysmenorrhea such as prevalence, duration and intensity of pain, risk factors, and methods used to reduce pain. Another objective was to evaluate the impact of dysmenorrhea on the quality of life by using our own specific questionnaire (the DysmenQoL questionnaire) that estimated the negative effects of dysmenorrhea on health and feelings, daily activities, relationships with others and professional activity. We also checked the validity and reliability of this specific questionnaire in our population.

We evaluated dysmenorrhea and its impact on the quality of life in participants of reproductive age and we did not limit the study to adolescents. We did not find in the literature a score or a specific questionnaire to observe the relationship between this condition and quality of life.

## 2. Materials and Methods

We conducted a cross-sectional study between January and April 2024 in Bucharest, Romania. In this study were included women of reproductive age between 18 and 45 years of age who agreed to participate in the study and who answered all of the questions. Women who did not meet the age criterion or who did not complete the entire form were not included in this study. The exclusion criteria were represented by women without menstruation, menopausal women, patients with diagnosed gynecological pathology (such as endometriosis, uterine fibromatosis, ovarian cysts or pelvic inflammatory disease), patient with previous gynecological surgeries, patients with medical or mental disorders or patients in their menstrual period at the time of distribution of the questionnaire.

This study received the ethical approval of our institution Saint John Hospital Bucharest (protocol code 9083/12 April 2023). The patients’ informed consent for participation in the study was obtained through online methods.

We collected data from the participants with our original form consisting of 71 questions: 36 questions with two or more answer options, 6 multiple choice questions, 9 questions with open answers and 20 statements scored from 1 to 5. This form was divided into three sections. It was structured with the online tool Google Forms and it was distributed through social media to specific groups of women.

The first section (27 questions) aimed to collect data about general characteristics: professional activity, medical history, diet, physical activities, lifestyle and menstrual period. Quantitative limits were defined for the frequent consumption of coffee (250 mL at least 3 times a week) and chocolate (30 g at least 3 times a week). All participants in the study answered the first section. The rest of the questions were addressed exclusively to the participants who stated that they have dysmenorrhea.

The second section (24 questions) aimed to collect data about the characteristics of dysmenorrhea: frequency, duration, location, intensity of menstrual pain and symptoms associated with it and methods used to reduce pain and evaluate their effectiveness. The intensity of the menstrual pain was evaluated by giving a number from 1 to 10 (1—no pain; 10—unbearable pain). Menstrual cycles were considered regular if they occurred at an interval between 21 and 35 days, and irregular if they did not respect this interval [21].

These two sections were useful in achieving the objective of evaluating the characteristics of dysmenorrhea (prevalence, risk factors, pain assessment).

The third section was useful for evaluating the impact of dysmenorrhea on the quality of life of the participants.

The last section was named “DysmenQoL questionnaire” (dysmenorrhea and quality of life) and it was represented by 20 statements about the negative effects of dysmenorrhea on quality of life (effects on health and feelings, relationships with family members or friends, couple relationships and professional activity). Only the study participants who stated that they have dysmenorrhea responded to this section. The twenty statements were developed after extensive consultation with clinicians, discussions with patients and literature review [22,23,24]. The participants gave a score from 1 to 5 for each statement: 1—“Never”; 2—“Few cases”; 3—“Sometimes”; 4—“Most of the time”; 5—“Every time”. We calculated the sum of the scores for each statement and we called it “DysmenQoL score”, whose value could vary between 20 and 100.

In addition, we checked the validity and reliability of our own DysmenQoL questionnaire in our population.

The database was created and analyzed using SPSS Statistics, version 23. We used descriptive statistics methods to calculate frequencies, means and standard deviations. We performed the statistical analysis by using tests corresponding to the types of variables and the proposed objectives: Pearson correlation, independent samples *t*-test, one-way ANOVA and Chi-squared. The results of these tests were statistically significant if the *p* value was below 0.05. We tested the validity and reliability of DysmenQoL questionnaire. Reliability was evaluated by calculating Cronbach’s alpha, which had to be above 0.7 for the score to be reliable. Validity was tested for each individual statement using Pearson correlation.

## 3. Results

In this study were included 504 participants aged between 18 and 45 years. The mean age was 30.95 ± 6.37 years. There were no participants who did not meet the inclusion criteria. The general characteristics of the participants were summarized in Table 1. The prevalence of dysmenorrhea among the participants included in the study was 83.7% (422 participants). The age of menarche varied between 8 and 18 years, with an average of 12.64 ± 1.42 years. Using the independent samples *t*-test, we found out that the average age of menarche was statistically significantly higher in the case of participants who do not have dysmenorrhea (*p* = 0.035).

Possible risk factors for the presence of dysmenorrhea were evaluated (Table 2). It was observed that dysmenorrhea was statistically significantly associated with the degree of menstrual bleeding (*p* = 0.017), the presence of dyspareunia (*p* = 0.002), infertility (*p* = 0.034), a family history of dysmenorrhea (*p* < 0.001), premenstrual syndrome (*p* < 0.001) and a history of gastritis (*p* = 0.040). The presence of dysmenorrhea did not have a statistically significant correlation with smoking status, the number of hours of sleep, the type of food eaten, coffee or chocolate consumption and the type of physical activity practiced.

A total of 312 participants (73.9%) considered that dysmenorrhea affected their quality of life. From the total of 422 participants with dysmenorrhea, 54.7% of the participants stated that dysmenorrhea appeared from the first menstruation and in 61.8% of the cases, it started from the first day of menstruation. A percentage of 64.9% of the participants stated that dysmenorrhea occurred with every menstruation, and 26.5% of the participants stated that the pain occurred during more than half of the periods. In 95% of the cases, the pain was located at the level of the pelvis and lower abdomen and in 40% of the cases, it radiated most frequently at the lumbar region. In 82.2% of the cases, menstrual pain lasted at least one day.

It was found that 65.4% of the participants considered that the intensity of the pain was greater if they had a stressful period, and 80.8% of the participants stated that the intensity of the pain did not decrease after the start of sexual life. Out of the 162 participants who gave birth at least once, 65 participants stated that after giving birth the characteristics of the menstrual pain changed.

The participants used both pharmacological and non-pharmacological methods to reduce pain in 69.9% of the cases. The most used pharmacological methods were NSAIDs (54.7%). Regarding the moment of starting the medication, 54.5% of the participants said that they start the medication when the pain becomes unbearable, 38.6% of the participants said that they start the medication when the pain appears and 13.3% of the participants said that they start the medication before pain occurs when they have scheduled events. Regarding the usefulness of the drugs, 65.9% of the participants stated that the drugs were useful most of the time in reducing menstrual pain, and 18.5% of the participants believed that if they take drugs before the onset of the pain, it will have a low intensity regardless of the number of days of medication. Drugs were considered useful in performing daily activities in normal conditions by 79.1% of the participants. The most used non-pharmacological methods were applying liquids or warm objects on the abdomen (53.6%), sleeping (42.4%), massaging painful areas (22.5%) and eating sweets (14.2%).

Menstrual pain was not the only disturbing symptom present during menstruation; it was also accompanied by agitation or irritability (54.0%), fatigue (63.5%), headaches (47.9%), diarrhea (38.2%), nausea (35.3%) and dizziness (32.0%).

The intensity of menstrual pain was considered by 313 participants (74.2%) the most important characteristic of pain for the negative effects on the quality of life. Most of the participants (72.3%) stated that work from home was useful during the menstrual period.

Each participant with dysmenorrhea evaluated the intensity of the pain giving a number from 1 to 10 (1—no pain; 10—unbearable pain). The mean pain intensity was 7.13 ± 1.78. In addition, they completed a section (the DysmenQoL questionnaire) with 20 affirmations with a score from 1 to 5 about the negative effects of dysmenorrhea on the quality of life. The DysmenQoL score was calculated by adding these twenty answers. The values of the DysmenQoL score varied between 22 and 100, and the mean score was 61.39 ± 16.61. Information about the relationship between these scores and pain intensity is presented in Table 3.

**Figure 1:**
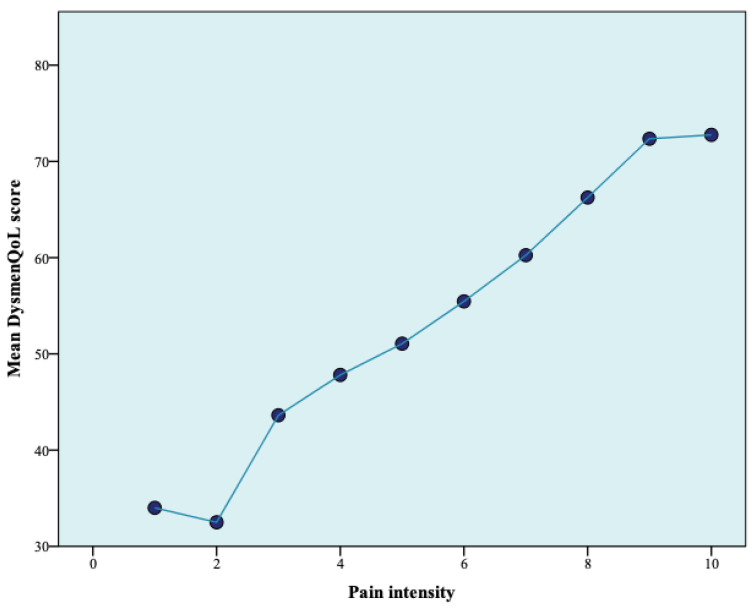
The relationship between pain intensity and DysmenQoL score.

Using the independent samples *t*-test, the patients with dysmenorrhea were divided into two groups: those who considered that dysmenorrhea affected their quality of life and those who claimed the opposite. It was observed that there was a statistically significant difference between these two groups regarding the mean pain intensity (*p* < 0.001) and the mean DysmenQoL score (*p* < 0.001). A statistically significant correlation was found between the pain intensity score and the DysmenQoL score (*p* < 0.001). The relationship between them was illustrated in Figure 1. No statistically significant correlation was observed between the DysmenQoL score and the age of the study participants (*p* = 0.344).

We used the one-way ANOVA test to analyze the DysmenQoL score. The results showed that there were statistically significant differences regarding the DysmenQoL score depending on the frequency (*p* < 0.001) and duration (*p* < 0.001) of dysmenorrhea, the intensity of pain (mild, moderate, severe) (*p* < 0.001), the methods used to reduce pain (*p* < 0.001) and the characteristic of pain that the participants considered the most important in affecting their quality of life (*p* < 0.001). No significant differences were observed regarding pain location (*p* = 0.116).

We used the independent samples *t*-test and found statistically significant differences regarding the DysmenQoL score and the presence of symptoms associated with menstrual pain: agitation or irritability (*p* = 0.009), nausea, vomiting, fatigue, diarrhea, headaches, dizziness, sweating, insomnia, loss of appetite for food (*p* < 0.001) and polyuria (*p* = 0.022).

The analysis of the scores for each statement is summarized in Table 4. It was observed that the average scores were increased for the following statements: “You feel more stressed” (3.75 ± 1.09), “You feel more agitated or nervous” (3.92 ± 0.99), “You feel more tired” (3.99 ± 0.99) and “You have less energy for daily activities” (4.04 ± 1.11). The lowest average was for the statement “Family members do not understand your discomfort and are not there for you” (2.01 ± 1.29).

We tested the reliability of this questionnaire (consisting of 20 statements) by calculating Cronbach’s alpha, which had a value of 0.928, which indicated an excellent internal consistency. We used the Pearson correlation to test the validity of this questionnaire and confirmed that each statement that contributed to the calculation of the DysmenQoL questionnaire was valid (*p* < 0.001). The DysmenQoL questionnaire proved to be a reliable and valid method for evaluating the impact of dysmenorrhea on the quality of life.

## 4. Discussion

We conducted a cross-sectional study that included 504 participants who fully answered our own questionnaire about dysmenorrhea. We evaluated the prevalence, risk factors and characteristics of menstrual pain. The questionnaire included a section dedicated to the impact of dysmenorrhea on the patients’ quality of life and based on the answers we calculated a score that we called the “DysmenQoL score”.

We found a high prevalence of dysmenorrhea (83.7%) among the participants included in the study. We identified a number of factors associated with the presence of dysmenorrhea, such as a familial history of dysmenorrhea and the presence of premenstrual syndrome, infertility or dyspareunia, as well as the degree of menstrual bleeding. Most of the participants believed that dysmenorrhea negatively affects their quality of life through the negative impact on health, affectivity, daily activities or relationships with others. The DysmenQoL score was useful in quantifying these negative effects. We hope that future studies will prove its effectiveness. Menstrual pain is a condition that requires more attention from those who provide health care because many women face this condition every month, for several days.

Dysmenorrhea is one of the most common gynecological complaints worldwide and can be found in over 90% of women of reproductive age [25,26]. In our study, the prevalence of dysmenorrhea was 83.7%.

A number of risk factors for the occurrence of dysmenorrhea were reported in the literature, such as a young age of menarche and a family history of dysmenorrhea and heavy menstrual flow [27]. Our data analysis indicated the same results. It has been reported that there is a positive relationship between the presence of dysmenorrhea and smoking, emotional problems, regular coffee consumption, physical activity [28], frequency of menstrual cycles [29], nulliparity [30], underweight, excessive sugar intake [31] and skipping breakfast [32]. In addition, we found that dyspareunia and infertility were associated with the presence of dysmenorrhea. Protective factors for the occurrence of dysmenorrhea were identified as older age, high fruit and vegetable consumption [33], young age at the time of first childbirth and the use of combined oral contraceptives [34].

In our study, 73.9% of the participants stated that dysmenorrhea affected their quality of life. In 54% of the cases, the participants stated that they presented agitation or irritability, and in 63.5% of these cases, they felt fatigue in the presence of dysmenorrhea. A total of 165 participants (39.1%) considered that the menstrual pain had a severe intensity. The mean pain intensity in this case was 8.69 ± 1.00, and the mean DysmenQoL score was 69.53 ± 15.90. Moderate pain was experienced by 230 participants (54.5%), with a pain intensity of 6.36 ± 1.22 and a DysmenQoL score of 57.34 ± 14.45. In a study conducted by Helwa et al., the average pain intensity among all participants was 6.79 ± 2.64 and most of the participants presented moderate or severe pain (80.34%) [24]. In another study by Ullah et al., it was observed that moderate meat or protein consumption and high levels of stress were associated with moderate or severe menstrual pain. The average intensity of dysmenorrhea among all study participants was 5.62 ± 2.28 and 65.8% of them had moderate or severe menstrual pain [35].

Dysmenorrhea is a public health problem worldwide. It was estimated that in the United States, approximately 600 million hours were lost from work annually due to dysmenorrhea [36]. In a study by Armor et al. which included 4202 teenage girls and young women, it was observed that absenteeism and concentration problems were more frequent during the menstrual period; almost half of the participants stated that they had missed a class in the last three menstrual periods and severe menstrual pain was associated with a decrease in academic performance [37]. A study by Schoep et al. included 42,879 women and assessed the impact of menstrual symptoms on quality of life. It has been observed that during the menstrual period 1 in 3 women cancel their daily activities due to the symptoms associated with menstruation [38]. It was found that the presence of dysmenorrhea could affect social life and relationships with family members and friends [39]. Intense menstrual pains negatively affect socializing, practicing physical exercises and personal study [22]. Hashim et al. used SF-36 (36-item Short-Form Health Survey) scales to quantify the impact of dysmenorrhea on quality of life and observed that domains related to emotional and physical health, including health changes and pain, were significantly negatively influenced by the presence of dysmenorrhea [40]. The results of another study that used the SF-36 indicated lower scores for the domains of general health, bodily pain and social function in the presence of dysmenorrhea [41]. Mizuta et al. used the World Health Organization Quality of Life scale and showed that the lowest quality of life scores were associated with severe dysmenorrhea [42]. It has been observed that dysmenorrhea can negatively affect the quality of sleep [43]. However, the polysomnogram performed during the menstrual periods did not highlight important changes in the sleep patterns of women who had menstrual pain [44].

The participants in our study stated that in 69.9% of cases, they use both pharmacological and non-pharmacological methods to reduce menstrual pain. NSAIDs were the most used drugs. The application of liquids or warm objects on the abdomen, sleeping or massaging painful areas represented frequently used non-pharmacological methods. Most of the participants considered that the administration of drugs was useful for reducing menstrual pain and for carrying out daily activities in normal conditions.

The persistence of menstrual pains and the negative effects that they have on patients can cause an exaggerated increase in drug consumption. Thus, it is very important to evaluate the intensity of dysmenorrhea and its impact on patients’ quality of life. It is necessary for women to learn to properly manage menstrual pain in safe conditions.

There are numerous dedicated questionnaires that evaluate the impact of gynecological symptoms or pathologies on quality of life, such as the ICIQ-VS for vaginal symptoms [45,46,47,48], P-QOL for genital prolapse [49,50,51], SQOL-F for quality of sexual life [52,53,54], FertiQoL for fertility problems [55,56], FACIT-CD for cervical dysplasia [57,58], PISQ-IR for the assessment of sexual function in patients with pelvic floor disorders [59,60,61], UFS-QOL for uterine fibromatosis [62,63] and MENQOL for the impact of symptoms associated with menopause [64,65]. We did not find a specific questionnaire for evaluating the impact of dysmenorrhea on quality of life. Future studies are required to reproduce the validity of the DymenQoL questionnaire.

The DysmenQoL questionnaire proved to be a specific, reliable, valid and useful tool in evaluating the impact of dysmenorrhea on quality of life. Its validity was supported by its statistically significant correlation with a series of dysmenorrhea characteristics such as duration, frequency and intensity (evaluated both quantitatively with a score from 1 to 10 and qualitatively—mild, moderate, severe). We believe that future studies will be necessary to confirm its use in other populations. We believe that this tool will prove useful in the evaluation of the therapeutic management of dysmenorrhea.

The limitations of our study were represented by the questions with predefined answers included in our questionnaire as well as by the accuracy of the answers of the participants in the study. Other limitations of the study were the possibility of misinterpretation of questions and the lack of interest and involvement of the participants during the response to the form, as well as the large number of questions.

## 5. Conclusions

The presence of dysmenorrhea was directly associated with the degree of menstrual bleeding, the presence of dyspareunia and infertility and the presence of premenstrual syndrome and a family history of dysmenorrhea. Most women considered that dysmenorrhea affected their quality of life, and this was correlated with the pain intensity and the DysmenQoL score. A significant difference regarding the DysmenQoL score depending on the pain intensity, frequency and duration of dysmenorrhea and the methods used to reduce the pain was observed. The DysmenQoL questionnaire proved to be a reliable and valid method for evaluating the impact of dysmenorrhea on the quality of life. Future studies are required to reproduce its validity.

## Figures and Tables

**Table 1:** General characteristics of the participants.

Characteristics	Participants (%)
Body Mass Index (BMI)	
<18.5 kg/m^2^	50 (9.9%)
18.5–25 kg/m^2^	308 (61.1%)
>25 kg/m^2^	146 (29.0%)
Studies	
High school studies	106 (21.0%)
Bachelor’s studies	215 (42.7%)
Master’s studies	161 (31.9%)
Doctoral studies	10 (2%)
Secondary school studies	12 (2.4%)
Monthly income	
<700 euro	96 (19%)
700–1000 euro	158 (31.3%)
1000–2000 euro	153 (30.4%)
>2000 euro	43 (8.5%)
Without a job	54 (10.7%)
Marital status	
Married	275 (54.6%)
Unmarried	222 (44%)
Divorced	6 (1.2%)
Widow	1 (0.2%)
Professional activity	
Entirely at the company office	317 (62.9%)
Hybrid system (at the company office and in the “remote” system—“work from home”)	72 (14.3%)
“Remote” system (“work from home”)	50 (9.9%)
Without a job	65 (12.9%)
Number of births	
0	342 (67.9%)
1	115 (22.8%)
2	46 (9.1%)
3	1 (0.2%)
Delivery	
Caesarean section	96 (19%)
Vaginal delivery	62 (12.4%)
At least one vaginal delivery and at least one cesarean section	4 (0.8%)
Not applicable	342 (67.9%)

**Table 2:** Risk factors for the presence of dysmenorrhea.

Characteristics	Dysmenorrhea (%)		Total (%)	χ^2^	* p *
	Yes	No			
Menstrual cycle				0.064	0.800
Regular (between 21 and 35 days)	345 (81.8)	68 (82.9)	413 (81.9)		
Irregular (<21 days or >35 days)	77 (18.2)	14 (17.1)	91 (18.1)		
Duration of menstruation				5.822	0.054
<3 days	19 (4.5)	4 (4.9)	23 (4.6)		
3–5 days	247 (58.5)	59 (59)	306 (60.7)		
>5 days	156 (37)	19 (23.2)	175 (34.7)		
Degree of bleeding				8.193	0.017
Normal bleeding (7–10 absorbents used)	258 (61.1)	56 (68.3)	314 (62.3)		
Heavy bleeding (>10 absorbents used)	104 (24.6)	9 (11)	113 (22.4)		
Less bleeding (<7 absorbents used)	60 (14.2)	17 (20.7)	77 (15.3)		
Sexual life				0.321	0.571
Active	344 (81.5)	69 (84.1)	413 (81.9)		
Inactive	78 (18.5)	13 (15.9)	91 (18.1)		
Combined oral contraceptives				3.045	0.081
Yes	28 (6.6)	10 (12.2)	38 (7.5)		
No	394 (93.4)	72 (87.8)	466 (92.5)		
Dyspareunia				14.868	0.002
Yes, most of the time	62 (14.7)	3 (3.7)	65 (12.9)		
Yes, in few cases	152 (36.0)	21 (25.6)	173 (34.3)		
No	186 (44.1)	53 (64.6)	239 (47.4)		
Not applicable	22 (5.2)	5 (6.1)	27 (5.4)		
Smoking				3.048	0.218
Yes	139 (32.9)	19 (23.2)	158 (31.3)		
Former smoker	73 (17.3)	16 (19.5)	89 (17.7)		
No	210 (49.8)	47 (57.3)	257 (51)		
Sleeping				1.584	0.453
<6 h	77 (18.2)	15 (18.3)	92 (18.3)		
6–9 h	337 (79.9)	67 (81.7)	404 (80.2)		
>9 h	8 (1.9)	0 (0.0)	8 (1.6)		
Premenstrual syndrome				96.405	< 0.001
Yes, most of the time	329 (78)	21 (25.6)	350 (69.4)		
Yes, in few cases	81 (19.2)	45 (54.9)	126 (25)		
No	12 (2.8)	16 (19.5)	28 (5.6)		
Family history of dysmenorrhea				51.658	< 0.001
Yes	295 (69.9)	23 (28.0)	318 (63.1)		
No	127 (30.1)	59 (72.0)	186 (36.9)		
Infertility				4.479	0.034
Yes	76 (18.0)	7 (8.5)	83 (16.5)		
No	346 (82.0)	75 (91.5)	421 (83.5)		
Gastritis				4.214	0.040
Yes	61 (14.5)	5 (6.1)	66 (13.1)		
No	361 (85.5)	77 (93.9)	438 (86.9)		
Frequent coffee consumption				0.861	0.354
Yes	319 (75.6)	58 (70.7)	377 (74.8)		
No	103 (24.4)	24 (29.3)	127 (25.2)		
Frequent chocolate consumption				0.352	0.553
Yes	190 (45.0)	34 (41.5)	224 (44.4)		
No	232 (55.0)	48 (58.5)	280 (55.6)		

**Table 3:** Pain intensity.

Characteristics	Pain Intensity		
	Mild	Moderate	Severe
Number of participants (%)	27 (6.4%)	230 (54.5%)	165 (39.1%)
Quality of life affected (%)			
Yes	9 (33.3%)	151 (65.7%)	152 (92.1%)
No	18 (66.7%)	79 (34.3%)	13 (7.9%)
Mean score of pain (±std *)	4.11 ± 1.28	6.36 ± 1.22	8.69 ± 1.00
Mean DysmenQoL score (±std)	46.03 ± 14.85	57.34 ± 14.45	69.53 ± 15.90

* std (standard deviation).

**Table 4:** DysmenQoL questionnaire.

Statements	Number of Participants (%) for Each Score					Mean ± Std
	1 Never	2 Few Cases	3 Sometimes	4 Most of the Time	5 Every Time	
1. “ You feel more agitated or nervous”	7 (1.7)	32 (7.6)	88 (20.9)	154 (36.5)	141 (33.4)	3.92 ± 0.99
2. “ You feel more tired”	7 (1.7)	28 (6.6)	85 (20.1)	145 (34.4)	157 (37.2)	3.99 ± 0.99
3. “ You feel more stressed”	16 (3.8)	39 (9.2)	107 (25.4)	132 (31.3)	128 (30.3)	3.75 ± 1.09
4. “ You have less energy for daily activities”	16 (3.8)	37 (8.8)	45 (10.7)	142 (33.6)	182 (43.1)	4.04 ± 1.11
5. “ You do not have adequate nutrition”	30 (7.1)	67 (23.0)	124 (29.4)	99 (23.5)	102 (24.2)	3.42 ± 1.21
6. “ You cannot practice your usual physical activities”	35 (8.3)	60 (14.2)	96 (22.7)	125 (29.6)	106 (25.1)	3.49 ± 1.24
7. “ The quality of sleep is altered”	47 (11.1)	70 (16.6)	117 (27.7)	99 (23.5)	89 (21.1)	3.27 ± 1.27
8. “ You change your schedule of vacations or recreational activities”	86 (20.4)	75 (17.8)	97 (23.0)	81 (19.2)	83 (19.7)	3.00 ± 1.40
9. “ You change your clothing style”	61 (14.5)	58 (13.7)	84 (19.9)	88 (20.9)	131 (31.0)	3.40 ± 1.41
10. “ You have conflicts with family members or life partner”	81 (19.2)	88 (20.9)	111 (26.3)	67 (15.9)	75 (17.8)	2.92 ± 1.35
11. “ You isolate yourself from family members or your life partner”	163 (38.6)	69 (16.4)	91 (21.6)	53 (12.6)	46 (10.9)	2.41 ± 1.38
12. “ You are less concerned with family duties”	119 (28.2)	87 (20.6)	108 (25.6)	65 (15.4)	43 (10.2)	2.59 ± 1.31
13. “ Family members do not understand your discomfort and are not there for you”	219 (51.9)	79 (18.7)	55 (13.0)	38 (9.0)	31 (7.3)	2.01 ± 1.29
14. “ Couple activities are affected”	102 (24.2)	101 (23.9)	105 (24.9)	66 (15.6)	48 (11.4)	2.66 ± 1.30
15. “ You avoid meetings with friends”	122 (28.9)	75 (17.8)	95 (22.5)	70 (16.6)	60 (14.2)	2.69 ± 1.40
16. “ You talk less with friends”	144 (34.1)	82 (19.4)	97 (23.0)	55 (13.0)	44 (10.4)	2.46 ± 1.35
17. “ You avoid professional discussions or meetings”	135 (32.0)	64 (15.2)	94 (22.3)	66 (15.6)	63 (14.9)	2.66 ± 1.44
18. “ You can not concentrate at work”	55 (13.0)	80 (19.0)	114 (27.0)	96 (22.7)	77 (18.2)	3.14 ± 1.28
19. “ You are not productive in carrying out professional activities”	59 (14.0)	78 (18.5)	107 (25.4)	97 (23.0)	81 (19.2)	3.15 ± 1.31
20. “ You have more conflicts or contradictory discussions with colleagues at work”	134 (31.8)	100 (23.7)	101 (23.9)	55 (13.0)	32 (7.6)	2.41 ± 1.26

## Data Availability

The data used in this study are available from the corresponding author, and the authors can share the information if there is a reasonable request.
